# Object size-dependent bias in aortic valve calcium quantification: polychromatic images vs virtual monochromatic images

**DOI:** 10.1186/s41747-026-00779-y

**Published:** 2026-08-14

**Authors:** Haruna Sagoh, Masaya Kisohara, Tsuyoshi Ito, Itsuki Honda, Nobuo Kitera, Seita Watanabe, Kazuma Murai, Takumi Fujinaga, Daisuke Takeichi, Ayano Kato, Akio Hiwatashi

**Affiliations:** 1 Department of Radiology, Nagoya City University Graduate School of Medical Sciences, Nagoya, Japan; 2 Department of Cardiology, Nagoya City University Graduate School of Medical Sciences, Nagoya, Japan; 3 Central Radiology Division, Nagoya City University Hospital, Nagoya, Japan; 4 Department of Radiology, Kasugai Municipal Hospital, Kasugai, Japan

**Keywords:** Aortic stenosis (calcific), Calcification of aortic valve, Multidetector computed tomography

## Abstract

**Objectives:**

To compare aortic valve calcification (AVC) quantifications between polychromatic images (PI) and 70-keV virtual monochromatic images (VMI) obtained from photon-counting detector CT (PCD-CT), and to assess the influence of object size on the quantifications.

**Materials and methods:**

This retrospective study included participants who underwent PCD-CT for aortic stenosis (AS) assessment or intervention planning between March 2023 and August 2025. AVC was quantified using aortic valve Agatston score (AVAS), calcium volume (AVCV), and calcium mass (AVCM) from both PI and 70-keV VMI. Wilcoxon signed-rank test assessed differences, and Spearman’s rank correlation coefficient and univariable linear regression analyses were used to evaluate associations between the PI/VMI ratio and water-equivalent diameter (WED). Agreement of AVAS-based classification between PI and 70-keV VMI was evaluated using Cohen’s κ.

**Results:**

A total of 137 participants (median age, 84 years [IQR, 79–88]; 81 women) were included. PI yielded significantly higher values than 70-keV VMI for AVAS, AVCV, and AVCM (all *p* < 0.001). The PI/VMI ratio showed a strong negative correlation with WED, particularly for AVCM (ρ = -0.73). In univariable regression analyses, WED showed negative associations with the PI/VMI ratios for AVCM and AVAS. Cohen’s κ for AVAS-based classification was 0.95–0.96.

**Conclusion:**

Compared with VMI-derived AVC quantification, PI-derived AVC quantification may be influenced by object size-dependent beam hardening. Although this did not lead to substantial AS-severity reclassification, differences in AVC quantification between PI and VMI may warrant consideration.

**Key Points:**

- **Question:** Do PI and VMI yield different AVC quantifications on PCD-CT?
- **Findings:** PI yielded systematically higher AVC volume, mass, and AVAS than 70-keV VMI. These differences may be associated with object size-dependent beam hardening.
- **Relevance statement:** Object size-dependent beam hardening may contribute to systematic differences in AVC quantification between PI and VMI. Although clinical reclassification was limited, reconstruction-specific differences may warrant consideration when applying CT-based thresholds for AS severity.

**Graphical Abstract:**

**Figure:**
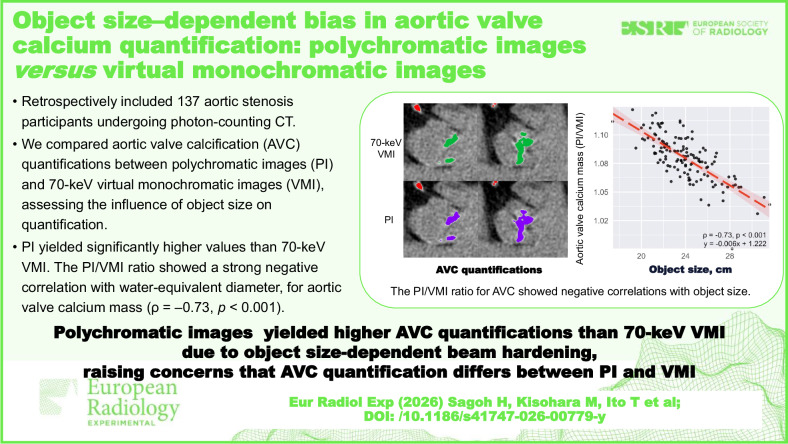

## Introduction

Photon-counting detector CT (PCD-CT) enables spectral imaging with reconstruction of both conventional polychromatic images (PI) and virtual monochromatic images (VMI) [1–3]. VMI has gained clinical adoption owing to its improved image uniformity and reduced beam-hardening artifacts [4, 5]. Previous studies, particularly in coronary artery calcium scoring, have reported numerical equivalence between VMI reconstructed at approximately 65–70 keV and PI acquired at 120 kVp [6]. However, these studies primarily evaluated numerical agreement and did not examine whether the equivalence remains stable across different patient object sizes. Because beam hardening in PI is inherently dependent on object size, the numerical consistency of PI-derived calcium metrics may vary across patients with different body habitus.

Aortic valve calcification (AVC) is a fundamental pathological feature of aortic stenosis (AS) and serves as a surrogate marker of disease severity when echocardiographic assessment is inconclusive [7]. CT-based AVC scoring—including the aortic valve Agatston score (AVAS), calcium volume (AVCV), and calcium mass (AVCM)—is integral to AS assessment and was originally validated using PI [8–12]. A critical limitation of PI is beam hardening, which induces object size- and tissue composition-dependent variability in measured CT number [13]. Consequently, AVC scoring derived from PI may be systematically influenced by patient object size, which is represented as water-equivalent diameter (WED) in the scan, potentially compromising numerical consistency of quantitative metrics. Despite this well-recognized behavior, widely used CT-based thresholds for defining severe AS—such as AVAS ≥ 2000 in men and ≥ 1200 in women [7, 9, 14]—implicitly assume numerical stability of Agatston-based measurements. These thresholds were established under PI conditions, yet their numerical interchangeability with VMI-derived measurements has not been systematically examined.

Based on prior experimental evidence indicating that beam-hardening effects are inherent to PI but substantially mitigated in VMI [5], we hypothesized that AVC metrics—AVCV, AVCM, and AVAS—exhibit systematic, object size-dependent differences between PI and VMI in both controlled phantom experiments and clinical data. Accordingly, this study aimed to evaluate whether object size affects AVC scoring in PI- and VMI-derived reconstructions.

## Methods

### Study patients

This retrospective single-center study was approved by the institutional review board (approval number: 60-25-0118), with a waiver of informed consent.

Study data were collected from a consecutive series of patients who underwent CT for transcatheter aortic valve implantation planning or assessment of AS severity with a dual-source PCD-CT scanner (NAEOTOM Alpha, Siemens Healthineers) from March 2023 to August 2025. Patients were eligible for inclusion if both PI and VMI could be reconstructed from the acquired CT data. Exclusion criteria were: (i) absence of a calcium scoring scan, (ii) scans performed with modalities other than PCD-CT, and (iii) lack of paired measurements derived from PI and VMI. AS severity was classified based on current clinical guidelines [15, 16].

### CT protocol

The scanning method was non-contrast electrocardiography-gated, with either axial or prospective helical scan selected. Scans were conducted at a tube voltage of 120 kVp, and automatic exposure control was applied using the CARE keV system with an IQ level of 19. Image reconstruction for PI and VMI at 50, 60, 70, 80, and 90 keV was standardized across all conditions, with a section thickness and increment of 3.0 mm, a Qr36 reconstruction kernel, and an iterative reconstruction strength of 2. Based on a previous report about coronary artery calcium scoring indicating a high degree of agreement between PI and VMI at approximately 70 keV, the main analysis was performed at 70 keV [6].

### Phantom study

To directly evaluate the influence of object size and reconstruction conditions on CT number independently of *in vivo* variability, a dedicated phantom experiment was performed. Three cylindrical anthropomorphic phantoms with different WEDs, representing small, medium, and large object sizes, were used. Supplementary Table S1 summarizes the manufacturers and dimensions of the phantoms. Each phantom was equipped with an identical calcium insert module containing calcified material with a physical density of 1.33 g/cm^3^ and an electron density relative to water of 1.266. All phantom scans were acquired using the same PCD-CT system and acquisition parameters as those used in the clinical study. For each reconstruction condition, mean CT number was measured using regions of interest placed at identical axial positions within the calcium insert. Measurements were repeated to account for variability. The effects of phantom size and reconstruction condition on CT number were evaluated using a linear mixed-effects model. Phantom size, reconstruction condition, and their interaction were included as fixed effects. To account for repeated measurements, a random intercept was included for each phantom size and slice combination.

### AVC scoring

AVC was quantified using dedicated software (syngo.via CT CaScoring, Siemens Healthineers). Although the software is primarily designed for coronary calcium scoring, two radiologists independently performed manual editing of the aortic valve calcium segmentation (M.K., board-certified radiologist with 10 years of experience in cardiovascular imaging; and K.M., radiologist in training with 4 years of experience). All included cases were evaluated by both readers. The readings were performed independently and in separate sessions, and both readers were blinded to each other’s results as well as to clinical and echocardiographic data. Interobserver agreement was assessed using intraclass correlation coefficients (ICCs). For subsequent analyses, measurements obtained by the more experienced reader (M.K.) were used. Measurements included AVCV, AVCM, and AVAS. The 130-HU threshold was intentionally kept constant to reflect current clinical calcium-scoring practice [10] and to evaluate real-world interchangeability between PI and 70-keV VMI rather than reconstruction-specific threshold optimization. This definition corresponds to the default threshold of this vendor’s software. The calibration factor for AVCM was 0.76 at PI and 70-keV VMI, 0.44 at 50-keV VMI, 0.60 at 60-keV VMI, 0.90 at 80-keV VMI, and 1.02 at 90-keV VMI. These calibration factors were specified by the vendor software and could not be adjusted. For manual segmentation, identical windowing settings were applied to both PI and VMI (window level: 30 HU; window width: 300 HU) to maintain consistency.

### Relation of AVC between PI and VMI

To evaluate the association between patient object size and differences in AVC quantification between PI and VMI, correlation analyses were performed between the PI/VMI ratio at each energy level and WED for AVCV, AVCM, and AVAS. Univariable linear regression analyses were performed using the logarithm of the PI/VMI ratio as the dependent variable and WED as the independent variable.

The numerical agreement of AS likelihood categories derived from PI and 70-keV VMI was evaluated using previously reported sex-specific AVAS thresholds. Classifications into categories such as “unlikely,” “unspecified,” “likely,” and “highly likely” were compared using confusion matrices. These categories were defined based on sex-specific AVAS values. For males, “Unlikely” was defined as AVAS < 1,600, “Unspecified” as 1,600 ≤ AVAS < 2,000, “Likely” as 2,000 ≤ AVAS < 3,000, and “Highly Likely” as AVAS ≥ 3,000. For females, “Unlikely” was defined as AVAS < 800, “Unspecified” as 800 ≤ AVAS < 1,200, “Likely” as 1,200 ≤ AVAS < 1,600, and “Highly Likely” as AVAS ≥ 1,600 [15, 17].

### Statistical analyses

Continuous variables were reported as mean ± SD or median with IQR, depending on distribution characteristics. Comparisons between PI-derived and VMI-derived values at each energy level for AVCV, AVCM, and AVAS were performed using the Wilcoxon signed-rank test. Spearman’s rank correlation coefficients were calculated to assess the strength of monotonic associations between variables. Passing–Bablok regression analysis was performed to evaluate the relationship between PI-derived and VMI-derived values at each energy level for AVCV, AVCM, and AVAS. Bland–Altman analyses were used to evaluate agreement and detect systematic biases between the two conditions. White’s test was employed to evaluate heteroscedasticity between measurements. Inter-rater reliabilities of AVC measurements were assessed with ICCs. ICCs were interpreted using the following thresholds: < 0.50, poor; 0.50–0.75, moderate; 0.75–0.90, good; and > 0.90, excellent agreement [18]. Univariable linear regression analyses were used to evaluate the association between WED and the logarithm of the PI/VMI ratio. Statistical significance was defined as a two-tailed *p* < 0.05.

Statistical analyses were conducted using Python version 3.9.19, incorporating the following open-source packages: numpy (version 1.26.4), scipy (version 1.13.1), scikit-learn (version 1.4.2), and statsmodels (version 0.14.2).

## Results

### Patient characteristics

**Fig. 1:**
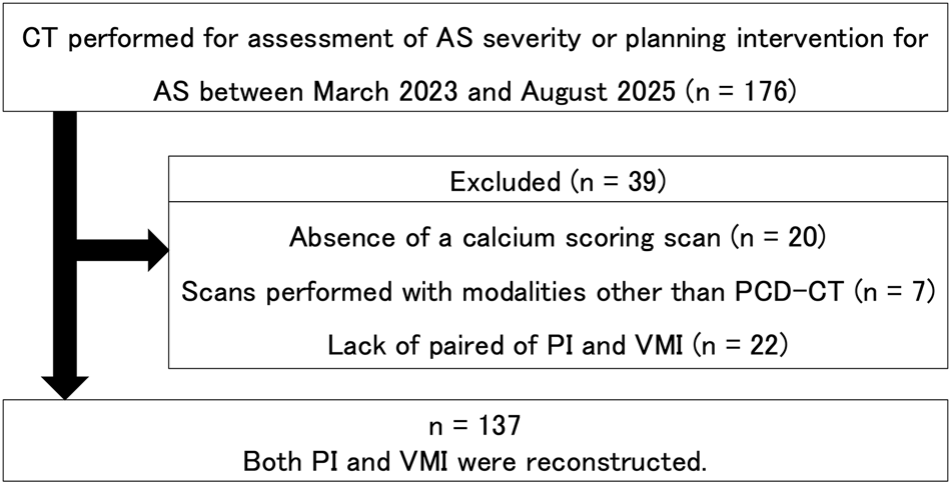
Flowchart of participant inclusion and exclusion. AS, Aortic stenosis; PCD-CT, Photon counting detector CT; PI, Polychromatic images; VMI, Virtual monochromatic images

During the study period, 176 participants underwent CT for AS assessment or treatment planning. The final study population comprised 137 participants (median age: 84 years [IQR: 79–88]; 81 were female) (Fig. 1). Participant demographics are summarized in Table 1.

**Table 1:** Participant characteristics

Demographics	Study population (*n* = 137), *n* (%)
Male, female	56 (41%), 81 (59%)
Age, years	84 [79–88]
BMI, kg/m^2^	22.5 [19.5–25.4]
Body weight, kg	54.1 [45.0–61.0]
Body surface area, m^2^	1.51 ± 0.18
Medical history	
Hypertension	100 (73%)
Hyperlipidemia	49 (36%)
Diabetes mellitus	36 (26%)
Angina or myocardial infarction	32 (23%)
Arrhythmia	38 (28%)
Heart failure	63 (46%)
Chronic kidney disease	37 (27%)
Echocardiographic findings	
Maximum velocity, m/s	4.3 [4.0–4.7]
Mean pressure gradient, mmHg	42.7 [35.2–54.9]
Dimensionless index	0.21 [0.17–0.27]
Aortic valve area, cm^2^	0.69 [0.55–0.82]
Left ventricular ejection fraction, %	65.0 [57.7–69.3]
Stroke volume index, mL/m^2^	30.3 [24.1–36.8]
Left ventricular end-diastolic volume index, mL/m^2^	49.0 [38.7–61.2]
Left ventricular end-systolic volume index, mL/m^2^	17.1 [12.9–24.8]
Left ventricular mass index, g/m^2^	110.6 [95.7–135.4]

Data are presented as mean ± SD or median [IQR] as appropriate. Body mass index is calculated as weight in kilograms divided by height in square meters

Echocardiographic findings are presented in Table 1. Based on clinical guidelines, AS severity was classified as severe in 73 participants, very severe in 31 participants, and moderate in 5 participants; no participants of mild AS were identified. Low-gradient discordant AS, defined by maximum aortic valve velocity < 4.0 m/s or mean pressure gradient ≤ 40 mmHg despite aortic valve area < 1.0 cm^2^, was observed in 28 participants.

The mean CT dose index volume was 2.5 ± 1.4 mGy, and the mean dose-length product was 39 ± 26 mGy·cm. The average WED was 23.9 ± 2.5 cm, and the mean heart rate during scanning was 69 ± 12 beats per minute.

### Phantom study

Supplementary Table S2 summarizes the results of the phantom experiment. In PI, mean CT number within the calcium insert decreased with increasing phantom size. In contrast, VMI showed markedly reduced size dependency across the evaluated energy range. Mean CT number decreased monotonically with increasing VMI energy for all phantom sizes, with inter-phantom differences remaining consistently smaller than those observed in PI. The estimated fixed effects from the linear mixed-effects model are shown in Supplementary Table S3. The reference condition (small phantom, PI) showed an estimated CT number of 529.5 HU. Compared with this reference, CT numbers decreased significantly with increasing phantom size in PI, with reductions of -15.6 HU for the medium phantom and -37.1 HU for the large phantom (both *p* < 0.001). Under VMI conditions, CT numbers demonstrated strong energy dependence. For the small phantom, CT numbers increased at lower energies (*e.g.*, +245.1 HU at 50 keV and +73.7 HU at 60 keV) and decreased at higher energies (*e.g.*, -32.1 HU at 70 keV, -99.8 HU at 80 keV, and -144.8 HU at 90 keV) relative to PI (all *p* < 0.001). Importantly, the magnitude of size-related differences was substantially smaller in VMI than in PI. For example, compared with the small phantom, the CT number differences in the large phantom ranged from +35.4 to +46.4 HU across all VMI energy levels, which was considerably smaller than the corresponding reduction observed in PI. A significant interaction between phantom size and reconstruction condition was observed (*p* < 0.001), indicating that the effect of object size on CT number differed according to reconstruction method. These findings demonstrate that VMI mitigates size-dependent variability in CT number compared with PI. The variance components of the random effects are summarized in Supplementary Table S4, showing that variability attributable to phantom–slice combinations (SD, 1.97 HU) and residual variability (SD, 2.01 HU) were both small relative to the observed fixed effects.

### AVC scoring

**Fig. 2:**
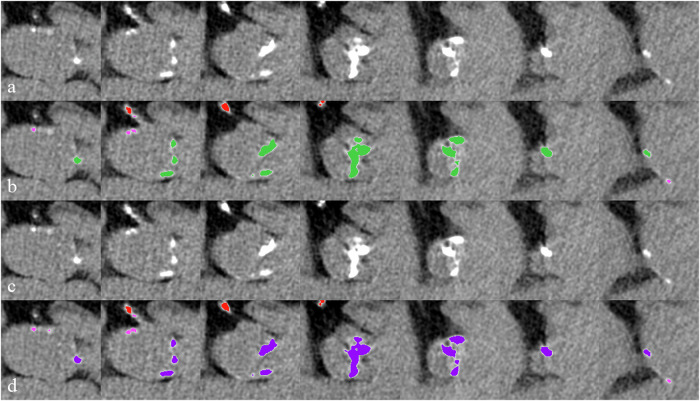
Quantification of AVC. **a** Presents the pre-analysis 70-keV VMI. **b** Displays the post-analysis 70-keV VMI. Green regions indicate segments of AVC. **c** Shows the pre-analysis PI. **d** Displays the post-analysis PI. Purple regions indicate segments of AVC. Pink and red regions indicate valid calcium pixels located at the aortic wall or the right coronary artery, respectively. PI, Polychromatic images; VMI, Virtual monochromatic images

Figure 2 presents a representative case of AVC segmentation on PI and 70-keV VMI. Although the difference was subtle, the region segmented on PI was slightly larger. The corresponding pixels on VMI did not exceed the threshold of 130 HU and were thus excluded from segmentation. No visual differences in CT number were observed in the calcified regions shared between the two conditions.

For all measured parameters, ICCs (2,1) values were greater than 0.99. Excellent inter-rater agreement was observed for AVCV, AVCM, and AVAS across all reconstruction conditions. Accordingly, subsequent analyses were conducted using AVCV, AVCM, and AVAS measurements obtained by the board-certified radiologist.

### Relation of AVC between PI and VMI

**Fig. 3:**
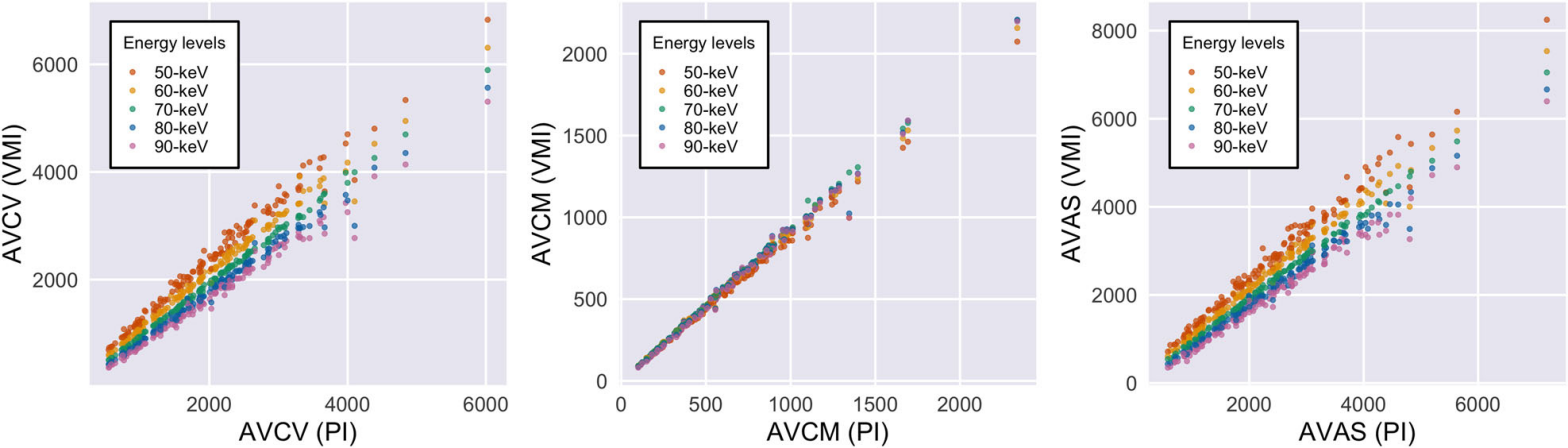
Correlation of AVCV, AVCM, and AVAS between PI and VMI at each keV level. There are significant correlations between PI and VMI at all keV levels for all three parameters (ρ > 0.99, *p* < 0.001). AVCV, Aortic valve calcium volume; AVCM, Aortic valve calcium mass; AVAS, Aortic valve Agatston score; PI, Polychromatic images; VMI, Virtual monochromatic images

Table 2 summarizes the median and IQR of AVCV, AVCM, and AVAS for each reconstruction condition. Within VMI reconstructions, AVCV and AVAS increased as the reconstructed energy decreased, reflecting higher CT numbers at lower keV levels. In contrast, AVCM demonstrated a different pattern because its calculation incorporates energy-specific calibration factors provided by the scanner. As a result, AVCM reached its highest value at 70-keV VMI. At energy levels of ≥ 70 keV, VMI-derived AVCV, AVCM, and AVAS were lower than PI. PI and VMI showed strong correlations across all keV levels for all parameters (all ρ > 0.99, all *p* < 0.001; Fig. 3). Passing–Bablok regression equations are summarized in Supplementary Table S5. For AVCV and AVAS, slopes were greater than 1.0 at lower energy levels (50–60 keV), indicating higher values on VMI than on PI, whereas slopes decreased below 1.0 at higher energy levels (≥ 70 keV), indicating lower values on VMI. In contrast, AVCM consistently showed slopes below 1.0 across all energy levels, indicating systematically lower mass values on VMI compared with PI. Among the evaluated energy levels, 70-keV VMI showed the smallest deviation from PI for AVCV and AVAS.

**Table 2:** Median and IQR of AVCV, AVCM, and AVAS for each reconstruction condition

	PI	50-keV VMI	60-keV VMI	70-keV VMI	80-keV VMI	90-keV VMI
AVCV, mm^3^	1,900 [1,260–2,560]	2,290 [1,490]	2,020 [1,530–3,020]	1,830 [1,230–2,480]	1,690 [1,080–2,310]	1,570 [1,000–2,180]
AVCM, mg	550 [330–820]	480 [300–730]	500 [300–760]	510 [310–770]	500 [300–770]	490 [290–760]
AVAS	2,240 [1,480–3,020]	2,660 [1,770–3,570]	2,390 [1,590–3,200]	2,160 [1,450–2,930]	1,980 [1,270–2,760]	1,870 [1,170–2,580]

*AVCV* Aortic valve calcium volume, *AVCM* Aortic valve calcium mass, *AVAS* Aortic valve Agatston score, *PI* Polychromatic images, *VMI* Virtual monochromatic image

**Fig. 4:**
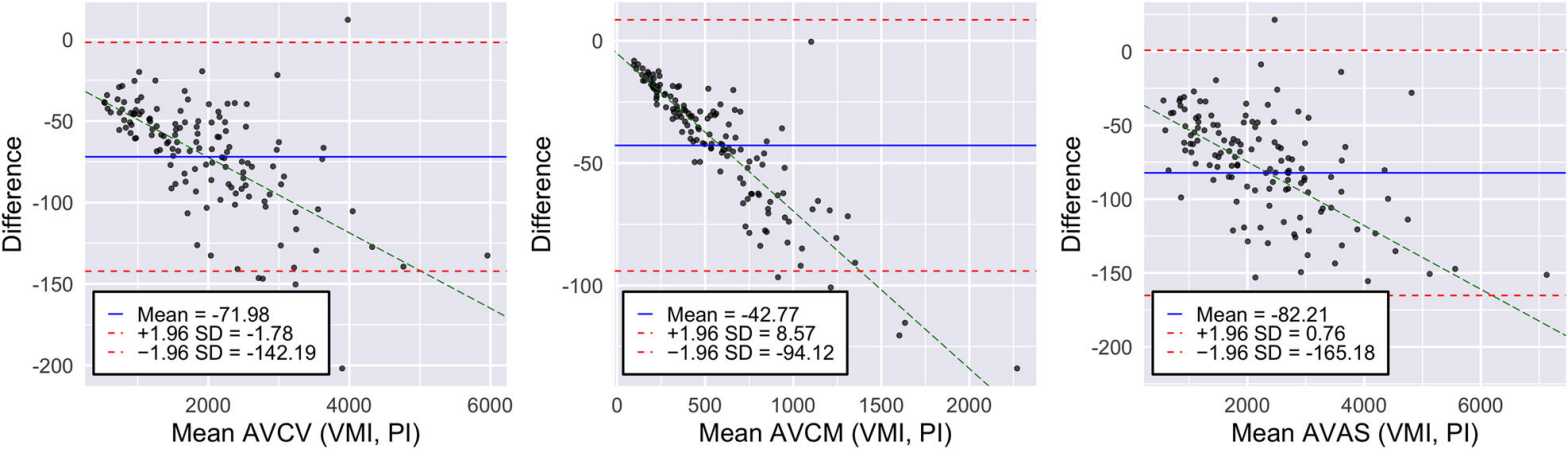
Bland–Altman plots of AVCV, AVCM, and AVAS between 70-keV VMI and PI. Blue solid line shows the mean of the difference. Red dashed lines show upper and lower limits of agreement. Proportional bias was observed in all parameters. Linear regression analysis demonstrated a significant association between the inter-method difference and the mean value (all *p* < 0.001). The strength of the association varied across parameters, with *R*² of 0.12 for AVCV, 0.72 for AVCM, and 0.13 for AVAS. The slope was negative in all cases, indicating that the difference became more negative with increasing mean values. AVCV, Aortic valve calcium volume; AVCM, Aortic valve calcium mass; AVAS, Aortic valve Agatston score; PI, Polychromatic images; VMI, Virtual monochromatic images

**Fig. 5:**
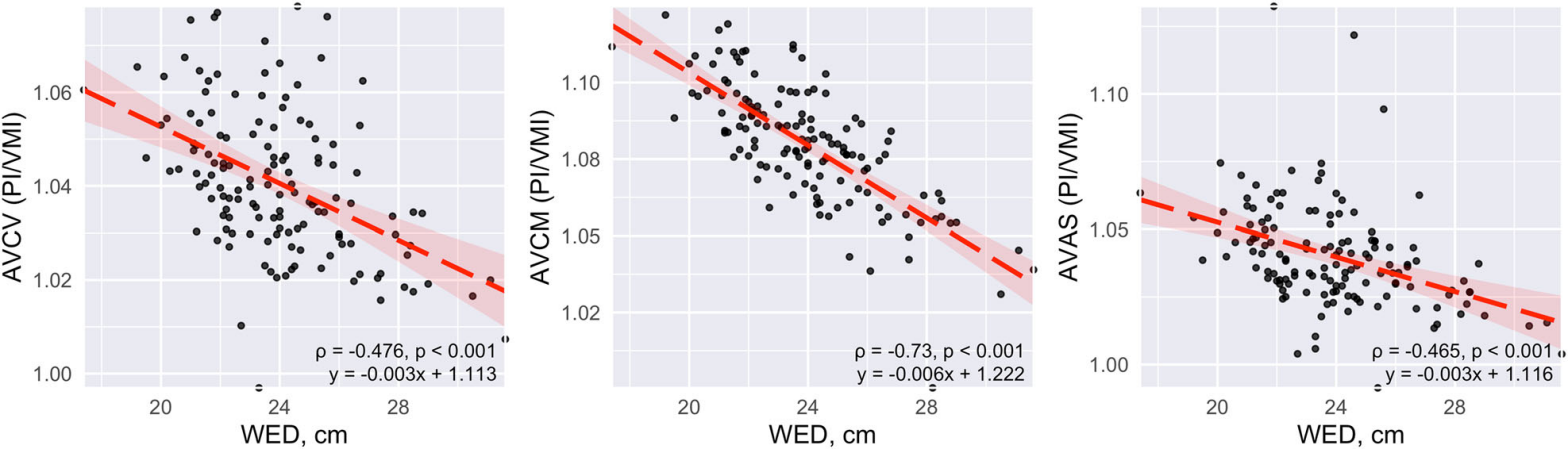
Correlation between the PI/70-keV VMI ratio and WED for each parameter. AVCV shows a Spearman’s rank correlation coefficient (ρ) of -0.48 (*p* < 0.001). AVCM shows ρ = -0.73 (*p* < 0.001). AVAS shows ρ = -0.46 (*p* < 0.001). AVCV, Aortic valve calcium volume; AVCM, Aortic valve calcium mass; AVAS, Aortic valve Agatston score; PI, Polychromatic images; VMI, Virtual monochromatic images; WED, Water-equivalent diameter

These differences were consistent in both sexes in the severe group (all *p* < 0.001). Similar differences were observed in other severity groups. There was no significant difference in the moderate or very severe groups in males, likely due to the small sample size (Table 3). Bland-Altman analyses demonstrated proportional bias across all parameters, indicating that the difference between PI and 70-keV VMI increased with the magnitude of the measurement (Fig. 4). White’s test revealed significant heteroscedasticity between the two conditions for AVCV (*p* < 0.001), AVCM (*p* = 0.002), and AVAS (*p* < 0.001), indicating non-constant variance between paired measurements. The association between WED and PI/VMI ratios was most consistent at 70-keV, with the strongest correlation observed for AVCM (ρ = -0.73), followed by AVCV (ρ = -0.48) and AVAS (ρ = -0.46) (all *p* < 0.001; Fig. 5).

**Table 3:** AVCV, AVCM, and AVAS by AS severity

Severity	Sex	AVCV (PI), mm^3^	AVCV (VMI), mm^3^	AVCM (PI), mg	AVCM (VMI), mg	AVAS (PI)	AVAS (VMI)
Moderate (*n* = 5)	M	1,840 [1,120–2,610]	1,760 [1,060–2,510]	610 [300–940]	570 [280–890]	2,180 [1,340–3,080]	2,080 [1,260–2,960]
	F	590	550	120	100	690	610
Severe (*n* = 73)	M	2,230 [1,870–2,570]	2,160 [1,780–2,520]	650 [510–870]	600 [470–820]	2,620 [2,210–3,070]	2,540 [2,060–3,020]
	F	1,280 [960–1,790]	1,240 [910–1,700]	340 [220–480]	310 [200–440]	1,490 [1,090–2,100]	1,460 [1,050–1,990]
Very severe (*n* = 31)	M	2,970 [2,730–3,180]	2,900 [2,650–3,100]	990 [930–1,040]	930 [880–960]	3,570 [3,260–3,800]	3,480 [3,160–3,720]
	F	2,490 [2,020–3,290]	2,340 [1,930–3,150]	770 [560–1,030]	690 [520–950]	2,960 [2,420–3,930]	2,840 [2,300–3,780]
Low gradient (*n* = 28)	M	2,100 [1,610–2,920]	1,970 [1,540–2,840]	670 [400–820]	650 [370–770]	2,460 [1,870–3,510]	2,480 [1,810–3,430]
	F	990 [830–1,300]	950 [780–1,230]	240 [220–330]	220 [200–300]	1,180 [970–1,520]	1,130 [900–1,450]

Continuous variables are presented as median [IQR]. Single values are presented because only one female patient had moderate AS. AS severity categories were defined based on echocardiographic criteria and do not necessarily correspond directly to CT-derived calcium burden; therefore, overlap between categories may occur

*M* Male, *F* Female, *AVCV* Aortic valve calcium volume, *AVCM* Aortic valve calcium mass, *AVAS* Aortic valve Agatston score, *AS* Aortic stenosis, *PI* Polychromatic images, *VMI* 70-keV virtual monochromatic image

Univariable linear regression analyses showed negative regression coefficients for WED across all metrics, although statistical significance varied across metrics (Table 4). Specifically, WED showed a consistent and statistically significant negative association with the AVCM ratio across all VMI energy levels. In contrast, for AVCV and AVAS, a similar negative trend was observed; however, statistical significance was reached only at selected energy levels (60 keV for AVCV and 70 keV for AVAS). Across different VMI energy levels (50–90 keV), the magnitude and direction of the association between WED and the PI/VMI ratio were consistent, with the strongest association observed for AVCM at 70 keV. The conversion formula from 70-keV VMI to the corresponding PI-derived metrics is provided in the supplementary materials.

**Table 4:** Univariable linear regression analyses of PI/VMI ratios and WED

	AVCV			AVCM			AVAS		
	β (95% CI)	*R*^2^	*p*	β (95% CI)	*R*^2^	*p*	β (95% CI)	*R*^2^	*p*
50 keV	-0.007 (-0.016, 0.003)	0.014	0.16	-0.012 (-0.018, -0.006)	0.11	< 0.001	-0.005 (-0.015, 0.005)	0.009	0.28
60 keV	-0.006 (-0.013, < 0)	0.030	0.043	-0.012 (-0.018, -0.007)	0.14	< 0.001	-0.006 (-0.013, > 0)	0.023	0.078
70 keV	-0.006 (-0.012, > 0)	0.025	0.065	-0.013 (-0.016, -0.009)	0.26	< 0.001	-0.007 (-0.013, -0.002)	0.044	0.013
80 keV	-0.006 (-0.014, 0.002)	0.018	0.11	-0.011 (-0.017, -0.005)	0.095	< 0.001	-0.008 (-0.017, 0.002)	0.018	0.12
90 keV	-0.007 (-0.018, 0.004)	0.012	0.21	-0.011 (-0.018, -0.004)	0.066	0.0025	-0.009 (-0.024, 0.006)	0.010	0.24

For β and the 95% confidence interval (CI), a value of “> 0” refers to a coefficient greater than zero, with a minimum of 0.001, while “< 0” refers to a coefficient less than zero, with a maximum of -0.001. β means the coefficient in univariable regression analysis, representing the change in the dependent variable when the independent variable increases by one

*CI* Confidence interval, *PI* Polychromatic images, *VMI* Virtual monochromatic image, *AVCV* Aortic valve calcium volume, *AVCM* Aortic valve calcium mass, *AVAS* Aortic valve Agatston score, *WED* Water-equivalent diameter

Finally, to assess the numerical concordance of the two conditions in classifying AS severity, participants were categorized into four likelihood levels—“Unlikely,” “Unspecified”, “Likely”, and “Highly likely”-based on sex-specific AVAS thresholds derived from PI. Agreement between conditions was high (Cohen’s κ = 0.95–0.96), indicating that the observed differences primarily reflected numerical shifts rather than widespread reclassification (Table 5). Three participants were classified as “Highly Likely” on PI but “Likely” on 70-keV VMI, and one participant was categorized as “Likely” on PI but “Unspecified” on 70-keV VMI. The results of the reclassification analysis after excluding patients with low-flow low-gradient AS are provided in the supplementary materials. The number of reclassifications was similar.

**Table 5:** Confusion matrix of reclassification for assessment of likelihood of severe AS using AVAS

		PI			
		Unlikely	Unspecified	Likely	Highly likely
70-keV VMI	Unlikely	9	0	0	0
	Unspecified	0	29	1	0
	Likely	0	0	34	3
	Highly likely	0	0	0	61

*AS* Aortic stenosis, *AVAS* Aortic valve Agatston score, *PI* Polychromatic images, *VMI* Virtual monochromatic image

## Discussion

In this study, we evaluated differences in AVC quantification between PI and VMI reconstructed from PCD-CT. Although PI and VMI showed extremely strong correlations for AVCV, AVCM, and AVAS, PI consistently yielded higher values than 70-keV VMI. Bland–Altman analyses demonstrated proportional bias, indicating that the discrepancy increased with the magnitude of calcification. These differences were associated with patient object size, represented by WED, most consistently for AVCM. The phantom experiment confirmed that CT numbers measured on PI decreased with increasing phantom size, whereas VMI showed markedly reduced size dependency. Passing–Bablok regression demonstrated that VMI values exceeded PI at lower energy levels for AVCV and AVAS, whereas at higher energies VMI values were lower than PI. The regression slopes approached unity around 70 keV, consistent with previous reports suggesting numerical similarity between approximately 70 keV VMI and conventional 120 kVp imaging, although small systematic differences remained. In contrast, AVCM values were consistently lower on VMI across all energy levels, suggesting that metrics incorporating CT number are more sensitive to spectral differences between reconstruction methods.

In the present phantom study, we demonstrated that the difference between PI and VMI is strongly dependent on object size. While VMI provides attenuation values at a predefined energy level, PI represents a spectrum-weighted attenuation that is inherently influenced by beam hardening. As object size increases, low-energy photons are preferentially attenuated, resulting in spectral hardening and a shift of the effective energy toward higher values. Consequently, the CT number of PI decreases with increasing phantom size, and its effective energy becomes closer to that of higher-keV VMI. This leads to a systematic alteration in the difference between PI and VMI across energy levels, as reflected by the significant interaction effects observed in our model. These findings indicate that the discrepancy between PI and VMI should not be interpreted solely as a fixed reconstruction-dependent difference, but rather as a size-dependent phenomenon driven by spectral distortion. This suggests that calcium scoring using fixed HU thresholds, may be affected by object size and may not be directly comparable across different reconstruction methods.

From a clinical perspective, the numerical differences between PI and VMI resulted in minimal reclassification of AS likelihood categories in our cohort. Nevertheless, the systematic shift between reconstruction methods suggests that direct application of PI-derived thresholds to VMI measurements may introduce bias, particularly in cases near diagnostic thresholds. These findings highlight the importance of considering reconstruction-dependent characteristics when interpreting AVAS-based severity assessment.

Our findings are consistent with prior research demonstrating the advantages of VMI in AVC scoring. VMI has been shown to reduce blooming artifacts and mitigate overestimation of calcification size and CT number, thereby improving quantification of coronary and valvular calcifications [19]. In contrast, PI is more susceptible to beam-hardening effects, which can lower CT numbers and distort calcium scoring [20]. Importantly, conventional AVAS thresholds for severe AS—such as ≥ 2,000 in men and ≥ 1200 in women—were established using PI acquired on energy-integrating detector CT systems [7, 9, 14]. Because VMI is reconstructed from spectral data obtained with PCD-CT and exhibits different attenuation characteristics, PI-based thresholds cannot necessarily be directly applied to VMI-derived measurements. In the present cohort, which consisted predominantly of elderly Asian patients with relatively small body habitus, VMI values were slightly lower than PI even at approximately 70-keV, despite the strong numerical correlation between the two reconstruction methods. Given the observed inverse association between WED and the PI/VMI ratio, these differences are likely to be attenuated in populations with larger body habitus. In cohorts with higher WED, such as Western populations or broader screening-like populations with greater variability in body size, the discrepancy between PI and VMI measurements may be less pronounced. Nevertheless, the underlying mechanism of size-dependent beam hardening is expected to be consistent, suggesting that reconstruction-specific considerations remain relevant even if their magnitude varies across populations. Although these differences resulted in only limited reclassification of AS likelihood categories, they were primarily observed in patients with calcium scores near established diagnostic thresholds. Accordingly, the clinical impact of these discrepancies is likely modest for most patients but may be relevant in borderline cases. From a practical perspective, PI- and VMI-derived AVC measurements should be interpreted consistently within the same reconstruction framework, and direct interchange between these measurements should be avoided, particularly when values are close to established diagnostic thresholds. Taken together, these findings suggest that AVC quantification derived from PI and VMI is not strictly interchangeable. AVAS thresholds established for PI should therefore be applied with caution when interpreting VMI-derived measurements, particularly in cases near decision thresholds. Rather than retrofitting VMI-derived measurements to legacy PI-based benchmarks, future studies may need to establish reconstruction-specific reference thresholds for AVC quantification.

This study has several limitations. First, the study population consisted predominantly of patients with severe or very severe AS, with relatively few cases of mild or moderate disease. As a result, the present study was not designed to establish or validate diagnostic thresholds across the full disease spectrum, and the impact of reconstruction-dependent differences in earlier disease stages remains uncertain. Second, although the phantom experiment confirmed the size-dependent behavior of PI and the relative stability of VMI, phantom models cannot fully reproduce the complex geometry and heterogeneous composition of valvular calcification observed *in vivo*. Therefore, the magnitude of reconstruction-related differences may differ under clinical conditions. Third, the study was conducted using a single PCD-CT platform and reconstruction algorithm. Because both spectral reconstruction and calcium quantification may vary across vendors and implementations, the observed relationships may not be directly generalizable to other CT systems. Fourth, for AVCM measurements, energy-dependent calibration was determined by the vendor implementation and was not user-adjustable; therefore, vendor-specific calibration behavior may have contributed to the observed differences, and these findings should be interpreted as reflecting combined effects of beam hardening, reconstruction characteristics, and calibration rather than a single mechanism. Fifth, a fixed threshold of 130 HU was used for calcium detection across all reconstructions. Because CT number is influenced by both reconstruction method and object size, the use of a uniform threshold may have contributed to the observed discrepancies in calcium quantification. In particular, beam-hardening-related attenuation shifts affect voxel intensities at lesion boundaries, thereby influencing partial volume effects and the apparent extent of calcification. Consequently, threshold-dependent segmentation behavior and beam-hardening-related attenuation changes are intrinsically coupled and jointly affect all derived metrics, with varying sensitivity across indices. Because threshold selection itself depends on CT number, modifying the threshold would not isolate these effects but rather alter segmentation in a manner that reflects both attenuation shifts and boundary-related phenomena. Therefore, although alternative thresholds may change numerical agreement between reconstruction methods, such analyses would primarily represent parameter optimization rather than a mechanistic evaluation of reconstruction-dependent differences. Accordingly, we applied a uniform threshold to reflect current clinical practice and to enable consistent comparison between PI and 70-keV VMI, while acknowledging that part of the observed differences may be attributable to threshold-dependent effects. Finally, the observed differences are likely multifactorial, potentially reflecting not only object size-dependent effects such as beam hardening but also threshold-dependent segmentation behavior, blooming, partial volume effects, and energy-dependent calibration differences. These factors should be considered when interpreting reconstruction-specific differences in AVC quantification.

In conclusion, AVC quantification derived from PI and VMI reconstructed from PCD-CT showed systematic numerical differences, which may be related to object size-dependent beam hardening affecting PI. Although these differences rarely altered clinical classification in this cohort, PI-based AVC thresholds may require cautious interpretation when applied to VMI-derived measurements.

## Supplementary information

**Additional file 1: Table S1.** Manufacturers and dimensions of the phantom. **Table S2.** Mean CT number and standard deviations of the calcium insert module by phantom size and reconstruction conditions. **Table S3.** Estimated fixed effects from the linear mixed-effects model of CT number in the calcium insert module by phantom size and reconstruction conditions. **Table S4.** Variance components of the random effects in the linear mixed-effects model. **Table S5.** Passing–Bablok regression equations for AVCV, AVCM, and AVAS derived from the relationship between PI and VMI at each energy level.

## Data Availability

The datasets used and/or analyzed during the current study are available from the corresponding author on reasonable request.

## Abbreviations

AS: Aortic stenosis
AVAS: Aortic valve Agatston score
AVC: Aortic valve calcification
AVCM: Aortic valve calcium mass
AVCV: Aortic valve calcium volume
PCD-CT: Photon-counting detector CT
PI: Polychromatic images
VMI: Virtual monochromatic image
WED: Water-equivalent diameter
