# Supplementary material for: Object size-dependent bias in aortic valve calcium quantification: polychromatic images vs virtual monochromatic images
Source: Eur Radiol Exp. 2026 Aug 14;10:115. doi: 10.1186/s41747-026-00779-y (PMC13476407; doi:10.1186/s41747-026-00779-y)
Supplement: Supplementary file 1 [file 41747_2026_779_MOESM1_ESM.pdf]

Object Size–Dependent Bias in Aortic Valve Calcium Quantification: Polychromatic Images vs Virtual Monochromatic Images

Electronic supplementary material

Methods

Correction of VMI-derived quantitative values to PI-equivalent values

Regression analysis of the logarithm of the ratio of PI to VMI against WED was used to calculate the correction formulas for each metric.

Reclassification of patients without low-flow, low-gradient AS

We performed a reclassification analysis on cases excluding those with low-flow, low-gradient (LFLG) AS and calculated Cohen's κ.

Results

Correction of VMI-derived quantitative values to PI-equivalent values

The following are the respective correction formulas.

$$AVCV_{corrected} = AVCV_{VMI} * e^{0.109347 - 0.002905 * \mathrm{WED}}$$

$$AVCM_{corrected} = AVCM_{VMI} * e^{0.208785 - 0.005518 * \mathrm{WED}}$$

$$AVAS_{corrected} = AVAS_{VMI} * e^{0.112658 - 0.003076 * \mathrm{WED}}$$

Reclassification of normal flow low gradient and high gradient AS

Sixteen LFLG AS patients were excluded, and 121 patients were reclassified via $AVAS_{PI}$ and $AVAS_{VMI}$. Three participants were classified as "Highly Likely" on PI but "Likely" on 70-keV VMI, and one participant was categorized as "Likely" on PI but "Unspecified" on 70-keV VMI. Agreement between conditions was high (Cohen's κ = 0.95).

Supplementary Table

Supplementary Table S1. Manufacturers and dimensions of the phantom

	Small	Medium	Large
Company Name	Sun Nuclear	Kyoto Kagaku Co., Ltd.	Sun Nuclear
Dimensions (cm)	21 × 21	20 × 30	30 × 40

Supplementary Table S2. Mean CT number and standard deviations of the calcium insert module by phantom size and reconstruction conditions

	Phantom size		
	Small	Medium	Large
PI	529.6 ± 1.8	513.9 ± 2.3	492.3 ± 3.4
50-keV VMI	774.7 ± 2.4	783.9 ± 4.2	783.8 ± 5.5
60-keV VMI	603.3 ± 1.9	606.7 ± 2.7	607.7 ± 3.7
70-keV VMI	497.5 ± 1.7	497.3 ± 2.0	498.7 ± 2.8
80-keV VMI	429.8 ± 1.7	427.4 ± 2.0	429.2 ± 2.6
90-keV VMI	384.8 ± 1.7	380.9 ± 2.1	382.9 ± 2.6

Values are presented as mean ± standard deviation (HU).

PI = polychromatic images, VMI = virtual monochromatic image

Supplementary Table S3. Estimated fixed effects from the linear mixed-effects model of CT number in the calcium insert module by phantom size and reconstruction conditions

	Effect	β	95% CI	*p*-value
Intercept	Small, PI	529.5	528.5, 530.4	< 0.001
Size	Medium	-15.6	-16.9, -14.2	< 0.001
	Large	-37.1	-38.5, -35.8	< 0.001
Energy	50-keV VMI	245.1	244.5, 245.7	< 0.001
	60-keV VMI	73.7	73.1, 74.3	< 0.001
	70-keV VMI	-32.1	-32.8, -31.5	< 0.001
	80-keV VMI	-99.8	-100.4, -99.2	< 0.001
	90-keV VMI	-144.8	-145.5, -144.2	< 0.001
Interaction	Medium × 50-keV VMI	24.9	24.1, 25.8	< 0.001
	Medium × 60-keV VMI	19.1	18.2, 19.9	< 0.001
	Medium × 70-keV VMI	15.5	14.7, 16.4	< 0.001
	Medium × 80-keV VMI	13.3	12.4, 14.2	< 0.001
	Medium × 90-keV VMI	11.8	11.0, 12.7	< 0.001
	Large × 50-keV VMI	46.4	45.5, 47.3	< 0.001
	Large × 60-keV VMI	41.7	40.8, 42.5	< 0.001
	Large × 70-keV VMI	38.5	37.7, 39.4	< 0.001
	Large × 80-keV VMI	36.6	35.8, 37.5	< 0.001
	Large × 90-keV VMI	35.4	34.6, 36.3	< 0.001

β = estimated regression coefficient, CI = confidence interval, PI = polychromatic images, VMI = virtual monochromatic image

The reference categories were the small phantom and PI. Confidence intervals were calculated using a Wald approximation, and *p*-values were estimated using the Satterthwaite method.

Supplementary Table S4. Variance components of the random effects in the linear mixed-effects model

Random effect	SD (HU)	Variance
Phantom–slice (random intercept)	1.97	3.86
Residual (within phantom–slice)	2.01	4.02

The random intercept accounts for baseline differences in CT number across phantom–slice combinations.

SD = standard deviation

Supplementary Table S5. Passing–Bablok regression equations for AVCV, AVCM, and AVAS derived from the relationship between PI and VMI at each energy level.

	Energy levels, keV	Regression equation	Spearman ρ	*p*-value
AVCV	50	y = 1.12x + 118	> 0.99	< 0.001
	60	y = 1.04x + 42	> 0.99	< 0.001
	70	y = 0.98x - 25	> 0.99	< 0.001
	80	y = 0.92x – 72	> 0.99	< 0.001
	90	y = 0.88x – 108	> 0.99	< 0.001
AVCM	50	y = 0.88x + 3	> 0.99	< 0.001
	60	y = 0.91x + 1	> 0.99	< 0.001
	70	y = 0.93x – 3	> 0.99	< 0.001
	80	y = 0.93x – 7	> 0.99	< 0.001
	90	y = 0.93x – 12	> 0.99	< 0.001
AVAS	50	y = 1.11x + 134	> 0.99	< 0.001
	60	y = 1.04x + 47	> 0.99	< 0.001
	70	y = 0.98x – 26	> 0.99	< 0.001
	80	y = 0.93x – 101	> 0.99	< 0.001
	90	y = 0.90x - 150	> 0.99	< 0.001

In the regression equations, x represents values measured on PI and y represents corresponding values measured on VMI. PI = polychromatic images, VMI = virtual monochromatic image, AVCV = aortic valve calcium volume, AVCM = aortic valve calcium mass, AVAS = aortic valve Agatston score
